# Everyone is searching for it and those who acquired it enjoy better mental health: a latent profile analysis of Chinese adolescents’ meaning in life

**DOI:** 10.3389/fpsyg.2024.1480499

**Published:** 2024-11-13

**Authors:** Xiaohua Zhou, Lu Yu, Chen Deng, Yaoxiang Ren, Meng Du

**Affiliations:** ^1^Teaching and Learning Centre, Lingnan University, Kowloon, Hong Kong SAR, China; ^2^Department of Applied Social Sciences, The Hong Kong Polytechnic University, Kowloon, Hong Kong SAR, China

**Keywords:** meaning in life, adolescents, mental health, latent profile analysis, China

## Abstract

**Objective:**

One factor associated with rising rates of depression and anxiety among youth is a lack of meaning in life (MIL). The importance of living a meaningful and purposeful life cannot be overstressed, especially for adolescents who are in a critical life stage and have recently experienced a 3-year-long global health crisis, namely the COVID-19 pandemic. Although previous studies have examined adolescents’ MIL, the majority of them adopted a variable-centered approach. The present study adopts a person-centered approach to investigate the updated MIL among Chinese adolescents in the aftermath of the COVID-19 pandemic, the demographic factors related to profile classification, and the differences in mental health among adolescents in different MIL classes.

**Methods:**

A questionnaire assessing MIL, depression, anxiety, stress, and demographic information was administered to 1,196 Chinese adolescents (mean age = 13.07 ± 0.58; 44.73% were female individuals). A three-step latent profile analysis was conducted.

**Results and discussion:**

This study revealed that (1) adolescents can be categorized into three classes: high MIL, medium MIL, and low and searching MIL. (2) Adolescents from intact families with higher educated mothers were more likely to be classified as high MIL class rather than low MIL class. Furthermore, (3) adolescents with high MIL experienced the best mental health outcomes, those with medium MIL experienced moderate mental health, and those with low MIL, who were still searching for MIL, exhibited the poorest mental health. External support may be necessary in the search process for Chinese adolescents. Future research could explore the process of searching for MIL and identify the challenges adolescents encounter when developing their sense of MIL.

## 1 Introduction

A recent survey conducted by Harvard Graduate School revealed that 36% of young adults felt anxious and 29% felt depressed, with a major contributing factor being a lack of meaning and purpose in their lives (Making Caring Common, 2023). More than half of the young adults (58%) reported that there was a lack of “meaning or purpose” in their lives. “Many are ‘achieving to achieve’ and find little meaning in either school or work” (Making Caring Common, 2023, p. 2). A survey in China reported that 40% of university students felt their lives had no meaning (Xu, 2017). This phenomenon raises concerns that meaning in life (MIL) may not be a default state for youth. It has been reported that more than 80% of 10th graders in China are constantly seeking meaning and purpose in their lives (Lin et al., 2021). It is unclear whether they will ultimately find their MIL.

MIL is formed by a network of insights and interpretations of our life experiences, desires, and plans for future achievements. It serves as an integrating factor in our lives, connecting the threads between our efforts to pursue happiness, withstand distress, and attain transcendence (Steger, 2009). It instills in us the belief that our lives have significance and that we are not merely passing time. It has long been recognized as central to human life and existence (Emmons, 1999; Hicks and King, 2009; Frankl, 1990; Steger, 2013). On the one hand, MIL is positively associated with subjective wellbeing (Arslan and Allen, 2022), especially life satisfaction (Li et al., 2022; Lin and Shek, 2019). On the other hand, a lack of MIL leads to psychological distress, including anxiety (Jing and Ding, 2024), health-risk behaviors such as illicit drug use (Brassai et al., 2011), and suicidal tendencies (Lew et al., 2020).

To be more specific, MIL has two dimensions: the presence of MIL (PMIL) and the search for MIL (SMIL; Steger, 2009). The presence dimension reflects ‘the degree to which people experience their lives as comprehensible and significant and feel a sense of purpose or mission in their lives that transcends the mundane concerns of daily life’ (Steger et al., 2008, p. 661). The SMIL refers to an individual’s desire and efforts to search for and explore the meaning of life. It encourages people to seek new opportunities and challenges, and motivates them to understand and organize their experiences (Steger, 2009). The two dimensions represent distinct but interrelated aspects of MIL. The former emphasizes experiencing meaning in the present moment, while the latter focuses on actively searching for meaning to live a purposeful life.

The PMIL is more consistently reported as a protective factor against mental health issues, including anxiety and depression (Chen et al., 2021; Yu and Chang, 2021). However, the association between SMIL and mental health is less consistent (for a comprehensive review, see Li et al., 2022). Kiang et al. found that SMIL was negatively associated with wellbeing (e.g., self-esteem) because it is often accompanied by internal turmoil among adolescents from Latin, Asian, and European American backgrounds (Kiang and Fuligni, 2010; Kiang and Witkow, 2015). Li J. B. et al. (2019) found that SMIL was positively related to internalizing problems among Italian adolescents, but not Chinese adolescents. Lin et al. (2021) focused exclusively on Chinese adolescents and found that SMIL was positively associated with life satisfaction, self-esteem, and positive affect. However, it was negatively associated with negative affect only for adolescents who exhibited low levels of PMIL. More research into the roles of the PMIL and SMIL in relation to the mental health of Chinese adolescents is warranted.

Adolescence is a critical life transitional stage from childhood to adulthood. During this critical developmental stage, individuals explore their identities (Dahl et al., 2018), seek to understand the self and the world (Erikson, 1968), and typically experience changes in various domains of their lives, such as physiological, psychological, family, school, and peer domains (Erikson, 1968). During this transitional stage, the majority of adolescents are in the process of searching for MIL (Lin et al., 2021). However, we cannot assume that searching for MIL necessarily leads to the presence of MIL. This highlights the need for empirical studies on adolescents’ SMIL and PMIL.

Apart from internal developmental factors, the external environment can also influence adolescents’ MIL. For example, the uncertain state of the world has been identified as a contributing factor to the lack of meaning in life among young adults (Making Caring Common, 2023). Over the past few years, the COVID-19 pandemic has emerged as a global public health crisis and a stressful event. The fear and uncertainty caused by the COVID-19 pandemic have heightened concerns about health and the potential death of oneself and others (Trzebiński et al., 2020), which may impact adolescents’ MIL. While negative effects (e.g., anxiety and depression) were common during the pandemic among adolescents worldwide (Panchal et al., 2023), some scholars have reminded us of the possibility of a delayed impact of the pandemic. They argued that post-traumatic stress, the long-term consequences of social restrictions, and maladaptive responses to the “new normal” are worth the attention of researchers (Min et al., 2021). From this perspective, the impact of the pandemic on MIL may have continued after its conclusion. Therefore, it is necessary to examine MIL among adolescents in the post-pandemic period to gain an updated understanding of adolescents’ MIL in the aftermath of the pandemic.

Regarding the factors influencing adolescents’ MIL, sociodemographic factors are important. Previous research has identified several sociodemographic factors that are related to MIL. For instance, a study on Brazilian adults (mean age = 33 ± 15.01) found that sex is related to MIL. Male adults are more likely to be in the existential indifference group, which is characterized by a lack of meaningfulness and an experience of a crisis of meaning (Damásio and Koller, 2015). A study among Ghanaian adults (mean age = 40.84 ± 11.20) found that living standards predicted the PMIL (Fadiji et al., 2023). However, not many studies have focused on adolescents’ sociodemographic factors in terms of MIL, especially among Chinese adolescents. This is another gap that the present study aimed to fill. Although we acknowledge the importance of other contributing factors (e.g., prosocial behavior, Xie et al., 2023), investigating which sociodemographic factors are related to MIL could provide a quick screening tool to help identify adolescents who are experiencing low MIL and need support.

Another gap that the present study aimed to fill was methodological. In the decades of research on MIL, the majority of the studies have been variable-centered (Li et al., 2021). However, MIL is a personal framework. There is no universal concept of MIL that applies to everyone. One’s life meaning is individually constructed (Frankl, 1990) and is determined by one’s value system that gives purpose and meaning to one’s being (Wong, 1998). Recently, more studies on MIL have adopted a person-centered approach using latent profile analysis. He et al. (2024) and Zhao et al. (2023) conducted latent profile analyses among medical college students and nursing students in China. Ma et al. (2023) classified high school students into four classes based on their level of meaning in life: high meaning in life, medium meaning in life, low meaning in life, and searching for meaning in life. The last group scored high in searching for meaning in life but low in the presence of meaning in life. However, their study (2023) did not include sociodemographic factors when classifying the profiles. The present study used a three-step latent profile analysis (Asparouhov and Muthén, 2014) to analyze the profiles of MIL among Chinese adolescents, examine the related sociodemographic factors, and explore the relationship between MIL and mental health based on profile membership.

In summary, considering the critical stage of adolescence and the possible impact of the aftermath of the pandemic, the present study aimed to examine the profiles of MIL, the related sociodemographic factors, and the differences in mental health classes among Chinese adolescents. The specific research questions included the following:

1. What are the categories of MIL among Chinese adolescents?
2. Which sociodemographic factors are related to the classification of Chinese adolescents’ MIL?
3. Does Chinese adolescents’ mental health differ across different MIL classes?

## 2 Materials and methods

### 2.1 Participants and procedures

Participants were recruited from four secondary schools in the Greater Bay Area of South China through convenience sampling between April and June 2023. All students studying in Secondary 1 (Grade 7) at the four schools were invited to participate in the study. They were informed that their participation was voluntary and confidential. Written consent forms were obtained before they began the questionnaire. Completing the questionnaire typically took 10–15 min. A total of 1,951 students participated in the study. After excluding responses that failed an attention check item and those with missing values, 1,196 (mean age = 13.07 ± 0.58) students were included in the data analyses (Table 1).

**Table 1:** Sociodemographic background of the participants.

Age	Mean	SD					
(*N* = 1,196)	13.07	0.58					
Sex	Female	Male					
(*N* = 1,196)	535 (44.73%)	661 (55.27%)					
Intact family (*N* = 1,182)	Intact family	Non-intact family					
	1,059 (89.59%)	123 (10.41%)					
Local	Local	Immigrant					
(*N* = 1,179)	842 (71.42%)	337 (28.58%)					
Father’s education level	No formal education	Primary school	Junior secondary school (Grade 7–9)	Senior secondary school (Grade 10–12)-	College (Without a bachelor’s degree)	Bachelor’s degree	Master’s degree and above
(*N* = 1,042)	30 (2.88%)	114 (10.94%)	484 (46.45%)	227 (21.79%)	90 (8.64%)	89 (8.54%)	8 (0.77%)
Mother’s education level	No formal education	Primary school (Grade 1–6)	Junior secondary school (Grade 7–9)	Senior secondary school (Grade 10–12)-	College (Without a bachelor’s degree)	Bachelor’s degree	Master’s degree and above
(*N* = 1,031)	69 (6.69%)	153 (14.84%)	425 (41.22%)	215 (20.85%)	74 (7.18%)	91 (8.83%)	4 (0.39%)

The present study was approved by the institutional review board of the authors’ university (HSEARS20230327004).

### 2.2 Measures

#### 2.2.1 Meaning in life questionnaire

The participants’ MIL was assessed using an adapted Chinese version of the Meaning in Life Questionnaire (MLQ; Steger et al., 2006; Liu and Gan, 2010). The Chinese MLQ consisted of nine items (Cronbach’s alpha = 0.71) and two subscales: the presence of meaning (five items) and the search for meaning (four items). The participants responded to the items on a 7-point scale ranging from 1 (“absolutely untrue”) to 7 (“absolutely true”). Higher scores indicated a greater presence of meaning and a stronger search for meaning. In the present study, the Cronbach’s alpha value was 0.78.

#### 2.2.2 Depression anxiety stress scale—youth

The participants’ mental health was assessed using the Depression Anxiety Stress Scales - Youth (DASS-Y) version (Szabo and Lovibond, 2022), which was developed specifically to assess depression, anxiety, and stress among adolescents and children. We translated the scale into Chinese using a back-translation approach. The original Cronbach’s alpha values were 0.89 (depression), 0.84 (anxiety), and 0.84 (stress). In the present study, they were 0.90 (depression), 0.87 (anxiety), and 0.91 (stress; Table 2).

**Table 2:** Descriptive statistics of MIL and mental health.

	Mean (SD)	Range		Cronbach’s alpha
		Minimum	Maximum	
MIL				
PMIL	24.07(6.59)	5	35	0.80
SMIL	20.08(4.98)	4	28	0.76
Mental health				
Depression	2.65(3.96)	0	21	0.90
Anxiety	1.54(3.06)	0	21	0.87
Stress	5.32(5.49)	0	21	0.92

In addition to MIL and mental health, sociodemographic information was also collected, including age, sex, native/immigrant status, and parents’ education levels and marital status.

### 2.3 Statistical analyses

Data cleaning was conducted before the data analyses. To assess the students’ attention while completing the questionnaire, one attention item was included in the questionnaire. After excluding careless responses from the participants who did not answer the attention check correctly (*N* = 613) and those with missing values on the MLQ (*N* = 142), a total of 1,196 responses were retained. First, a three-step latent profile analysis was conducted to classify the participants’ MIL and to examine the associations between the different classes of MIL and sociodemographic factors. During the latent profile analysis, the following model fit indices were used: the Akaike information criterion (AIC), Bayesian information criterion (BIC), adjusted BIC (aBIC), entropy, and *p*-values for the Lo–Mendell–Rubin (LMR) test and bootstrapped likelihood ratio test (BLRT). Smaller values of the ACI, BIC, and aBIC indicate a better model fit. An entropy value closer to 1 indicates higher classification accuracy. *p*-values of less than 0.05 for the LMR test and BLRT indicate a satisfactory model fit (Asparouhov and Muthén, 2014). Furthermore, multivariate analysis of variance (MANOVA) was conducted to compare mental health (i.e., depression, anxiety, and stress) across the different MIL profiles. All data were analyzed using Mplus Version 8 and SPSS Version 23.0.

## 3 Results

### 3.1 Descriptive statistics

As self-report measures may produce common method bias (Zhou and Long, 2004), we first used Harman’s one-factor test to assess for common method bias. For the MILQ and DASS-Y assessments, a single factor accounted for 37.72% and 48.55%, respectively, which is considered acceptable for further analysis. The participants scored higher on the PMIL than the SMIL, and their stress levels were higher than anxiety and depression (Table 2).

### 3.2 Latent classes of MIL

For the latent profile analysis of MIL, the present study initially started with two classes and then increased the number of classes by one each time. The results showed that the AIC, BIC, and aBIC were decreasing. The *p*-value for the LMRT was greater than 0.05 when there were four classes. The percentage of class 1 was less than 5% when there were five classes. Therefore, overall, a three-class solution was deemed the best, despite having a lower than the four-class and five-class solutions (Table 3).

**Table 3:** Model indices for the latent profiles of meaning in life.

Class	AIC	BIC	aBIC	Entropy	LMRT *p*-value	BLRT *p*-value	Profile prevalence				
							1	2	3	4	5
2	40420.26	40473.82	40473.82	0.79	< 0.001	< 0.001	589 49.12%	610 50.88%			
**3**	**39772.32**	**39965.71**	**39845.01**	**0.85**	**< 0.001**	**< 0.001**	**600** **50.04%**	**139** **11.59%**	**460** **38.37%**		
4	39232.99	39477.28	39324.81	0.87	0.096	< 0.001	120 10.00%	96 8.01%	608 50.71%	375 31.28%	
5	38860.13	39155.31	38971.08	0.87	< 0.01	< 0.001	52 4.34%	119 9.92%	371 30.92%	566 47.21%	91 7.59%
6	21481.19	21684.76	21557.70	0.82	0.021	< 0.001	60 5.00%	108 9.01%	35 2.92%	307 25.61%	389 32.44%

Note: The selected solution, i.e., 3 three-class solution, is shown in bold.

**Figure 1:**
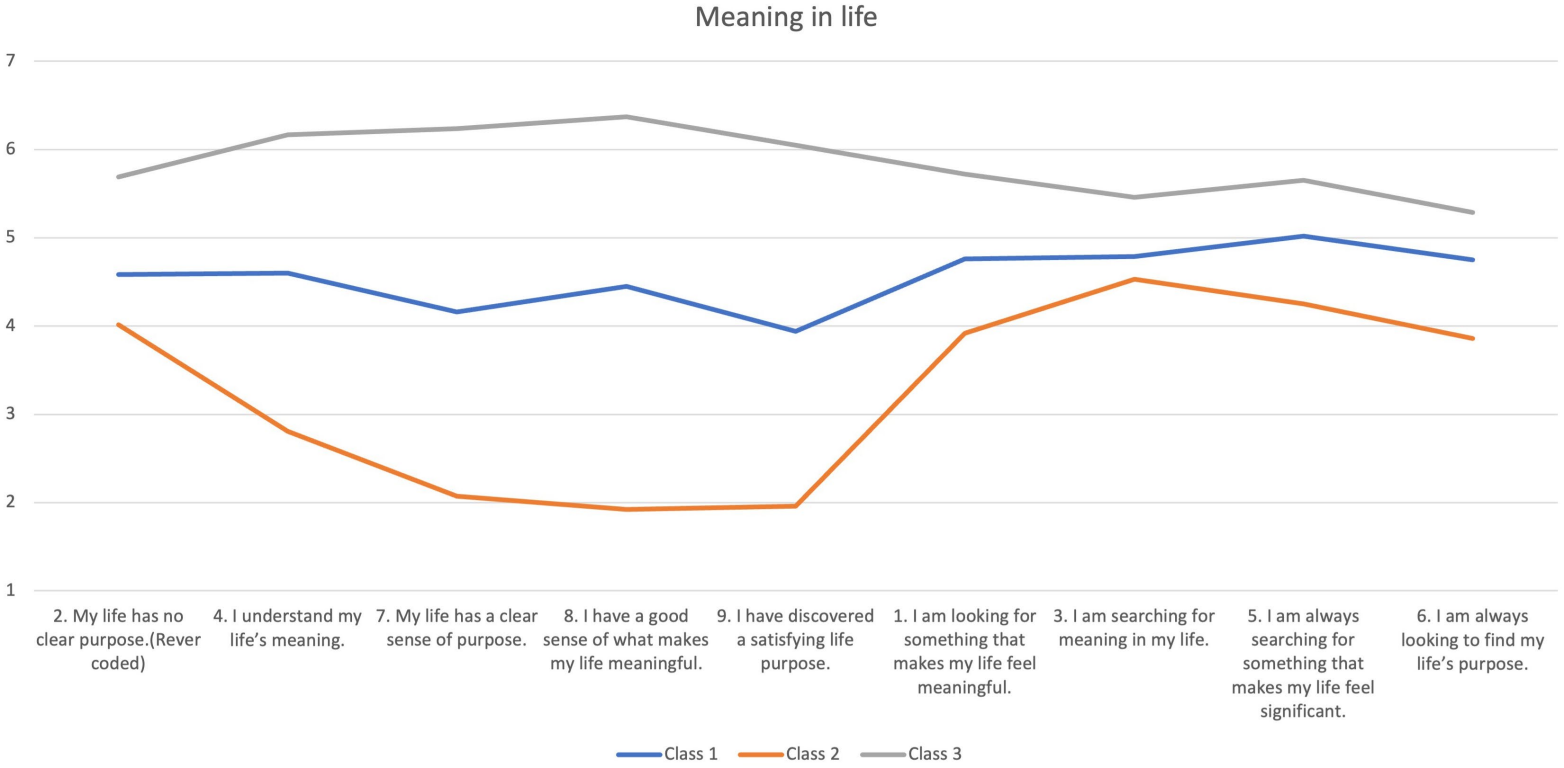
Three classes of MIL.

Class 1 represented a medium level of PMIL and SMIL and was therefore labeled “medium MIL.” Approximately half of the participants (*n* = 600, 50.04%) were searching for their MIL at a medium level and found a medium level of the presence of MIL. Class 2 represented a lower level of PMIL and SMIL compared to the other two classes, with the smallest percentage (11.59%, *n* = 139). The PMIL was lower than the SMIL, leading to its label as “low and searching MIL.” Within this class, the participants scored higher on the subscale of the SMIL and lower on the subscale of the PMIL. The remaining participants (*n* = 460, 38.37%) were categorized into Class 3 and scored higher on both PMIL and SMIL. Thus, Class 3 was labeled “high MIL.” Taking the three classes together, the differences (Table 4; Figure 1) in the PMIL were larger than those in the SMIL. This suggested that the participants were searching for MIL at a similar level; however, some of them found high MIL (Class 3), half of them enjoyed medium MIL (Class 1), while a small percentage of them had low MIL (Class 2).

**Table 4:** Comparison of the PMIL and SMIL among the three classes.

	Class 1 (Medium MIL) (*n* = 600)	Class 2 (Low and Searching MIL) (*n* = 139)	Class 3 (High MIL) (*n* = 460)	Comparisons
	Mean (SD)	Mean (SD)	Mean (SD)	
PMIL	4.35 (0.63)	2.56 (0.74)	6.11 (0.60)	Class 2 < Class 1 < Class 3
SMIL	4.83 (0.99)	4.14 (1.53)	5.53 (1.23)	Class 2 < Class 1 < Class 3

The differences between Class 1 and Class 2, Class 2 and Class 3, and Class 1 and Class 3 were all significant at the 0.001 level.

### 3.3 Sociodemographic factors

Regarding the sociodemographic factors associated with the MIL profiles, the results revealed that the participants whose mothers had higher education levels and those from intact families were 1.30 times and 3.21 times more likely, respectively, to be classified into Class 3 (high MIL) compared to Class 2 (low and searching MIL; Table 5).

**Table 5:** Odds ratios of the sociodemographic factors on the MIL profiles.

	Class 1 (Medium MIL)				Class 3 (High MIL)			
	OR	SE	95% CI		OR	SE	95% CI	
			Lower	Upper			Lower	Upper
Age	0.85	0.20	0.53	1.34	0.89	0.21	0.56	1.40
Sex (1 = Female)	1.13	0.28	0.70	1.83	0.70	0.17	0.44	1.13
Father’s education level	1.03	0.15	0.77	1.38	0.98	0.14	0.74	1.30
Mother’s education level	1.14	0.15	0.88	1.46	1.30	0.17	1.01	1.67
Intact family (1 = Intact family)	1.36	0.52	0.64	2.88	3.21	1.39	1.37	7.52
Local (1 = Local)	0.71	0.20	0.41	1.23	1.05	0.29	0.61	1.82

Reference group: Class 2 (Low and Searching MIL).

### 3.4 Mental health across the different classes of MIL

Based on the class membership that resulted from the latent profile analysis, this study examined whether the participants in the different classes experienced different mental health statuses, including depression, anxiety, and stress. The results revealed that the participants in the three classes reported significantly different levels of depression, anxiety, and stress. The participants in Class 3 (high MIL) reported the lowest levels of depression, anxiety, and stress, while those in Class 1 (medium MIL) reported moderate levels and those in Class 2 (low and searching MIL) reported the highest levels. The most significant difference was in depression (χ^2^ (2) = 69.75, *p* < 0.001; Table 6).

**Table 6:** Mental health comparison among different profiles.

	Classes	Mean	SE	Between class differences (class k versus class k + 1)		Overall
				C1	C2	
Depression	C1 Medium MIL	2.73	0.17	–		69.75^***^
	C2 Low and Searching MIL	5.65	0.48	30.44^***^	–	
	C3 High MIL	1.64	0.18	18.68^***^	61.15^***^	
Anxiety	C1 Medium MIL	1.64	0.14	–		37.57^***^
	C2 Low and Searching MIL	3.11	0.37	12.88^***^	–	
	C3 High MIL	0.94	0.13	12.43^***^	30.69^***^	
Stress	C1 Medium MIL	5.30	0.23	–		60.83^***^
	C2 Low and Searching MIL	9.20	0.62	32.39^***^	–	
	C3 High MIL	4.12	0.25	10.64^**^	58.15^***^	

^**^p < 0.01 and ^***^p < 0.001.

## 4 Discussion

### 4.1 Latent classes in MIL

In the present study, the participants generally had a good sense of meaning in life. Three latent classes were identified, namely “high MIL,” “medium MIL,” and “low and searching MIL.” This finding is largely consistent with Ma et al.’s (2023) study, in which high school students (mean age = 16.33 ± 1.01) were classified into four classes: “high MIL,” “medium MIL,” “searching MIL,” and “low MIL.” Both Ma et al.’s (2023) study and the present study classified Chinese adolescents based on their levels of MIL. Moreover, adolescents with medium and high levels of MIL accounted for a large percentage in both studies, indicating that the majority of adolescents in China reported medium and high levels of MIL.

Both studies exhibited a pattern of “high SMIL and low PMIL” within a single class: the “searching MIL” class in Ma et al.’s (2023) study and the “low and searching MIL” class in the present study, with the gap between high SMIL and low PMIL being smaller. The two studies revealed a similar phenomenon: Chinese adolescents were searching for MIL, but not everyone had found it. Unlike their peers in the “low MIL” class in Ma et al.’s (2023) study, who scored the lowest in both PMIL and SMIL groups, these adolescents were actively seeking something that would make their lives meaningful and significant, as well as trying to figure out their life purposes; however, they failed to find them. They may need external support in the process of searching for their MIL. For example, secondary schools could provide a life education curriculum to help these adolescents understand themselves and their life events, as well as explore their purpose and the meaning of their lives.

However, the pattern “high SMIL and low PMIL” was not reported in He et al.’s (2024) study conducted on college medical students (mean age = 21.73 ± 1.13) in China, which identified three classes through an LPA, and the PMIL was not lower than the SMIL. This may suggest that the phenomenon of adolescents searching for but failing to acquire MIL disappears when they enter early adulthood. Another possible reason is that He et al. (2024) conducted their study among medical students who might have encountered more opportunities to reflect on MIL during their studies (e.g., interacting with patients). This indicates that adolescents who are still in their searching phase may need more opportunities to be inspired by MIL.

### 4.2 Sociodemographic factors related to classification

The present study revealed that adolescents from intact families were more likely to be classified in the high MIL class compared to being classified in the low and searching MIL classes. This finding is consistent with the results of Shek et al.’s 2021 study, which reported that family intactness positively predicted MIL among adolescents. One possible explanation is that adolescents from intact families may have more resources and fewer challenges in their search for and acquisition of MIL. The attention and resources from both biological parents can help adolescents gain more life experiences, which is essential for searching for and acquiring MIL. This process requires life experience and the ability to interpret and make sense of what has happened in our lives (Steger, 2009). Parental support is related to MIL even among young adults through optimism and identity commitment (Kealy et al., 2022). On the contrary, adolescents from single-parent families and stepfamilies reported more suicidal thoughts (Garnefski and Diekstra, 1997), which may indicate a failure in searching for and acquiring their MIL.

The present study also found that the adolescents whose mothers had higher education levels were more likely to be classified in the high MIL class compared to being classified in the low and searching MIL class. One possible reason is that mothers with higher education levels may offer more opportunities for their children to explore the world and themselves, which are important for shaping one’s MIL. As for why mothers’ education levels, rather than fathers’, are related to classification in the high versus low and searching MIL classes, it may be because mothers play a larger role in childcare and parenthood (Shek et al., 2019; Zou et al., 2019). Therefore, mothers’ education levels play a more significant role than those of fathers.

The findings regarding the importance of both family intactness and mothers’ education levels are consistent with the literature review, which identifies the family as the most important source of meaning in people’s lives (Glaw et al., 2017).

### 4.3 MIL and mental health

The present study revealed that MIL was generally related to adolescents’ depression, anxiety, and stress. The adolescents with high MIL reported significantly less depression, anxiety, and stress. This finding is consistent with previous studies among adolescents in the US (Dulaney et al., 2018), Spain, and Russia (Shub et al., 2024), highlighting the cross-cultural importance of MIL as a protective factor for adolescents’ mental health. Moreover, this finding builds on Krok (2018), which demonstrated that MIL is positively correlated with subjective wellbeing and psychological wellbeing, including factors such as autonomy, environmental mastery, personal growth, positive relations with others, purpose in life, and self-acceptance. While Krok (2018) revealed a positive relation between MIL and the positive indicators of wellbeing, the present study revealed a negative relation between MIL and negative mental health issues, such as depression, anxiety, and stress. Krok (2018) also suggested that the purpose embedded in the concept of MIL appears central to the formation of wellbeing in late adolescents, whose mean age is approximately 18 years in Poland. This study also found that the central role of MIL in influencing mental health is true for early adolescents (age = 13.07 ± 0.58) in China. Overall, these findings indicate the importance of leading a meaningful and purposeful life in fostering good mental health and wellbeing holds true across different cultures and throughout both early and late adolescent years.

Another noteworthy observation is that the largest difference among the three categories of MIL was observed in depression rather than in stress or anxiety. This highlights the crucial role of MIL in addressing depression among adolescents. Finding out the purpose and meaning of their lives could protect adolescents from depression, a major health concern for them (Daly, 2022). Therefore, educators and social workers are encouraged to provide education, interventions, and support in the search for MIL to prevent depression among adolescents.

### 4.4 Contributions and limitations

Although the relationship between MIL and mental health has been examined before (for a literature review, see Glaw et al., 2017; Li et al., 2021), this study makes three unique contributions:

First, it employed a person-centered method instead of a variable-centered method. As mentioned earlier, searching for and acquiring MIL are individual experiences and endeavors. Therefore, a person-centered approach may be more appropriate for capturing the differences among individual adolescents. For instance, the latent profile analysis revealed a combination of relatively high SMIL and low PMIL in Class 2, which may not be easily identified using a variable-centered approach.

Second, the results revealed that the differences in mental health, such as depression, anxiety, and stress, were mainly caused by the PMIL rather than the SMIL among adolescents. This highlights that high SMIL does not necessarily guarantee high PMIL. Therefore, the intervention program should focus on helping adolescents find their MIL and identify a life purpose. For example, secondary schools can provide life education courses to enhance students’ understanding of life events, help them understand themselves, and encourage them to explore the spirituality of life.

Finally, this study was conducted in the aftermath of the COVID-19 pandemic, and the results indicated that even after a 3-year-long global health crisis, the majority of Chinese adolescents possess a relatively medium to high MIL.

The study has several limitations that should be recognized. First, as it was a cross-sectional survey, the adolescents with better mental health might have had more mental resources to focus on deep questions regarding the meaning and purposes of their lives, making them more likely to find their meaning in life. Second, the convenience sampling might have limited the generalizability of the findings of this study. In the future, including adolescents from different regions in China in the survey may enhance its representativeness. More importantly, while we gathered snapshot data on the SMIL and PMIL, searching for and acquiring MIL are not endpoint statuses. Understanding the processes of SMIL and changing PMIL among adolescents may yield more interesting findings.

## 5 Conclusion

In summary, by applying a person-centered approach, we categorized the participants into three classes: high MIL, medium MIL, and low and searching MIL. We found that the adolescents whose mothers had higher education levels and those who came from intact families were more likely to be classified in the high MIL class rather than the low and searching MIL class. Moreover, we found that the adolescents with high MIL experienced the best mental health outcomes, those with medium MIL experienced moderate mental health, and those with low MIL and who were still searching for MIL exhibited the poorest mental health. Intervention programs and secondary school curricula may need to focus on supporting the search process for MIL among Chinese adolescents. Future research could delve into the process of searching for MIL and identify the challenges that adolescents encounter when developing their sense of MIL.

## Data Availability

All data can be made available upon request to the corresponding author.
